# Comparative analysis of adeno-associated virus serotypes for gene transfer in organotypic heart slices

**DOI:** 10.1186/s12967-020-02605-4

**Published:** 2020-11-18

**Authors:** Zihou Liu, Kristin Klose, Sebastian Neuber, Meng Jiang, Manfred Gossen, Christof Stamm

**Affiliations:** 1 Berlin Institute of Health Center for Regenerative Therapies, Charité – Universitätsmedizin Berlin, Berlin, Germany; 2 Department of Cardiothoracic and Vascular Surgery, German Heart Center Berlin, Berlin, Germany; 3 German Centre for Cardiovascular Research, Partner Site Berlin, Berlin, Germany; 4 Berlin-Brandenburg Center for Regenerative Therapies, Berlin, Germany; 5 Helmholtz-Zentrum Geesthacht, Institute of Biomaterial Science, Teltow, Germany

**Keywords:** Myocardial slice, Organotypic heart slice culture, Adeno-associated virus, Gene transfer, Gene therapy

## Abstract

**Background:**

Vectors derived from adeno-associated viruses (AAVs) are widely used for gene transfer both in vitro and in vivo and have gained increasing interest as shuttle systems to deliver therapeutic genes to the heart. However, there is little information on their tissue penetration and cytotoxicity, as well as the optimal AAV serotype for transferring genes to diseased hearts. Therefore, we aimed to establish an organotypic heart slice culture system for mouse left ventricular (LV) myocardium and use this platform to analyze gene transfer efficiency, cell tropism, and toxicity of different AAV serotypes.

**Methods:**

LV tissue slices, 300 µm thick, were prepared from 15- to 17-day-old transgenic alpha-myosin heavy-chain-mCherry mice using a vibrating microtome. Tissue slice viability in air-liquid culture was evaluated by calcein-acetoxymethyl ester staining, mCherry fluorescence intensity, and the tetrazolium assay. Four recombinant AAV serotypes (1, 2, 6, 8) expressing green fluorescent protein (GFP) under the CAG promoter were added to the slice surface. Gene transfer efficiency was quantified as the number of GFP-positive cells per slice. AAV cell tropism was examined by comparing the number of GFP-positive cardiomyocytes (CMs) and fibroblasts within heart slices.

**Results:**

Slices retained viability in in vitro culture for at least 5 days. After adding AAV particles, AAV6-infected slices showed the highest number of GFP-expressing cells, almost exclusively CMs. Slice incubation with AAV1, 2, and 8 resulted in fewer GFP-positive cells, with AAV2 having the lowest gene transfer efficiency. None of the AAV serotypes tested caused significant cytotoxicity when compared to non-infected control slices.

**Conclusions:**

We have established a readily available mouse organotypic heart slice culture model and provided evidence that AAV6 may be a promising gene therapy vector for heart failure and other cardiac diseases.

## Background

Heart failure (HF) is one of the most important causes of morbidity and mortality worldwide. More than 26 million people suffer from HF globally, and its prevalence is still growing [1]. Despite improvements in previous decades of available medical treatments, the prognosis of HF remains poor, with an average 1-year mortality rate of 33% [2]. Although heart transplantation is the gold standard therapy for patients with end-stage HF, routine treatment is still challenging due to the increasing lack of donor organs, limited graft survival, and the long-term complications of immunosuppressive therapy. Gene therapy based on viral vectors is a promising new approach and offers great potential for the treatment of patients with end-stage HF [3, 4]. Among the multitude of available viral gene delivery systems, adeno-associated virus (AAV) vectors are appealing candidates because of their ability to mediate efficient transfer and stable expression of therapeutic genes in a wide variety of tissues, such as brain, liver, and heart [5–7]. AAVs have several advantages over other viral vectors. First, AAV naturally infects humans, inducing a very mild immune response but is not known to cause disease. Second, recombinant AAV vectors are able to induce long-term transgene expression in non-dividing cells after a single delivery. Third, as a gene therapy vector, AAV provides the ability to target a particular tissue or cell type by using a specific AAV serotype [8, 9]. Thirteen AAV serotypes and more than 100 AAV variants have been isolated from human/non-human primate tissues and evaluated in various model systems [10, 11]. Application of AAV vectors for gene transfer into the heart was first demonstrated more than 20 years ago [12], and significant progress has been made in the clinical translation of AAV-mediated cardiac gene therapy in recent years [13]. Among the different AAV serotypes, AAV1, 6, 8, and 9 have been identified as the most cardiotropic ones [5]; however data regarding their efficiency of cardiac gene transfer are inconsistent. Various studies have shown that AAV9 mediates highly efficient gene delivery to the heart [5, 14–16], whereas others have described AAV1, 6, and 8 as alternative cardiotropic vectors to AAV9 [17–24]. In more recent work, AAV6 was identified as an efficient serotype for the infection of stem cell-derived cardiomyocytes (CMs) [25]. Therefore, more evidence is required to determine the ideal AAV serotype for gene therapy in heart disease.

Organotypic heart slices are widely accepted as a powerful in situ platform for cardiovascular research and thus provide an excellent tool for studying AAV-mediated gene transfer into cardiac cells [26]. As multicellular models of myocardial tissue, they represent an intermediate between monolayer cultures and in vivo models, preserving the native cellular milieu and extracellular matrix network of the heart, the complex non-cellular three-dimensional architecture, as well as the physiology and pathology of the heart [27]. In studies involving human and animal tissue, heart slices with a thickness of 300 µm remained viable for several days under air-liquid interface culture conditions [28, 29]. Importantly, this model system has already been used to investigate adenovirus-mediated gene transfer [30, 31]. More recently, an optimized model of myocardial slices cultured under electromechanical stimulation has been described, in which the authors showed that an adenovirus encoding four cell cycle factors could induce CM proliferation [32]. To date, however, there are no reports on AAV-mediated gene delivery in organotypic heart slices.

In the present study, we aimed to investigate the gene transfer efficiency, expression kinetics, penetration depth, cell-type tropism, and cytotoxicity of AAV1, 2, 6, and 8 using a three-dimensional mouse heart slice culture model. The AAV serotypes were selected based on their different levels of transgene expression in vivo; for example, AAV1, 6, and 8 were described to mediate a strong gene expression in rodent hearts, whereas the non-cardiotropic AAV2 induced gene expression in this region relatively slowly [22]. AAV9 was not included in this study since it was not available at the time the study was performed. Finally, we sought to uncover the optimal serotype for targeting specific cell types in the heart, thereby accelerating the clinical translation of gene therapy for heart disease.

## Methods

### Animals

The following homozygous transgenic mouse line was used in this study: B6;D2-Tg(Myh6*-mCherry)2Mik/J (Jackson Laboratory, stock no. 021577), which is characterized by alpha-myosin heavy-chain (αMHC)-driven mCherry expression in CMs. Mice were obtained from the Research Facility for Experimental Medicine of the Charité – Universitätsmedizin Berlin. All mice were healthy and not subjected to any treatment or surgery before use. All procedures involving animals were conducted in accordance with the German Animal Welfare Act and the Charité Animal Welfare Guidelines. The study protocol was approved by the State Office of Health and Social Affairs Berlin (Registration no. T0158/15).

### AAVs

AAVs were obtained from the Viral Core Facility of the Charité – Universitätsmedizin Berlin and stored at − 80 °C until use. AAV serotypes used were as follows: AAV1 (no. BA-01 l), AAV2 (no. BA-01 h), AAV6 (no. BA-01 d), and AAV8 (no. BA-01 g); all of them drive green fluorescence protein (GFP) expression under the control of the CAG promoter, a hybrid of the cytomegalovirus early enhancer element and chicken beta-actin promoter. Recombinant AAVs used were hybrid AAV vectors that have been generated using the capsid protein of AAV1, 2, 6, and 8 and the genome of AAV2.

### Heart slice preparation

Mice at the age of 16 ± 1 days were sacrificed by cervical dislocation under isoflurane anesthesia. The chest was opened, the heart was excised, and the left ventricular (LV) tissue was isolated and stretched to be as flat as possible. The tissue was then embedded epicardial-side down in a premade 4% agarose gel block and glued onto the tissue holder of a high precision vibrating microtome (HM 650 V, Microm). The cutting chamber was connected to a cooling unit (CU 65, Microm) and filled with cold (4 °C) oxygenated (100%) Tyrode’s cutting solution (140 mM NaCl, 6 mM KCl, 10 mM Glucose, 10 mM HEPES, 1 mM MgCl2, 1.8 mM CaCl2, 30 mM BDM, pH 7.4). After the endocardium was trimmed off, myocardial slices were cut tangential to the epicardium with a thickness of 100–300 µm. The advance speed was 0.08 mm/s, and the vibration amplitude was set to 1 mm at a constant frequency of 80 Hz. To obtain uniformly sized tissues, slices were shaped using a 4 mm-diameter biopsy punch (pfm-medical).

### Heart slice culture

Before transferring the slices into culture, they were washed six times with Dulbecco's phosphate-buffered saline with magnesium and calcium (DPBS; Gibco, catalog no. 14040117) with 3% penicillin/streptomycin (P/S; Gibco, catalog no. 15140122). Slices were cultured at the air-liquid interface using porous transwell inserts (Millipore, catalog no. MCHT06H48), which were placed in 6-well plates with 1.5 ml Medium 199 (Gibco, catalog no. 41150020) containing 1% P/S and 1 × insulin-transferrin-selenium (Gibco, catalog no. 41400045). Plates with slices were placed in a humidified incubator set to 37 °C, 5% carbon dioxide and either 1% (hypoxia) or 20% (normoxia) oxygen. After 24 h in culture, slices were washed three times with DPBS supplemented with 3% P/S and the culture medium was changed. Thereafter, the medium was changed every 2 days.

### Heart slice viability analysis

Viability of organotypic heart slices was assessed using (i) calcein-acetoxymethyl ester (calcein-AM) staining, (ii) mCherry fluorescence intensity, and (iii) the 3-(4,5-dimethylthiazol-2-yl)-5-(3-carboxymethoxyphenyl)-2-(4-sulfophenyl)-2H-tetrazolium (MTS) assay. For the calcein-AM assay, heart slices were placed in 48-well plates with 200 µl culture medium containing 20 µM calcein-AM (MoBiTec, catalog no. MFP-C430) and incubated for 15 min at 37 °C. Living cells take up the non-fluorescent calcein-AM and hydrolyze it to the fluorescent calcein by intracellular esterases. Twenty µl of Hoechst 33342 (Thermo Scientific, catalog no. R37610) were added to the medium to visualize the whole slice by nuclear staining and slices were incubated for another 15 min at 37 °C. After washing three times with DPBS, images of both sides of slices were taken using a fluorescence microscope (Axio Observer Z1, Carl Zeiss). Brightness and contrast of images were adjusted using AxioVision (version 4.9.1.0) and Adobe Photoshop CC 2018 (version 19). Image analysis was conducted by measuring the area of calcein-stained cells using the tracing tool of the ImageJ software (version 1.48v). To determine the total mCherry fluorescence intensity per slice, images of heart slices were taken as described above and image analysis was performed by manual tracing in ImageJ following an established protocol [33]. For the MTS assay, MTS (Promega, catalog no. G5421) and the electron coupling reagent phenazine methosulfate (Sigma, catalog no. P9625) were mixed in a 20:1 (v/v) ratio, and 20 µl of this solution were added to 100 µl culture medium in 96-well plates. One myocardial slice was added to each well and incubated for 90 min at 37 °C. In the presence of metabolically active cells, MTS is reduced to the brightly colored but non-fluorescent formazan. One hundred and twenty µl of the supernatant were transferred to a new well and 30 µl dimethyl sulfoxide were added to each slice and incubated for an additional 70 min at 37 °C to lyse cells and solubilize the formazan crystals prior to measurement. The second supernatant taken was combined with the first one and mixed thoroughly. Absorbance was measured at 490 nm using a multi-well plate reader (SpectraMax 340PC, Molecular Devices).

### AAV infection and gene transfer analysis

AAV1, 2, 6, and 8 were tested. In detail, 4 × 10^8^ vector genomes of each serotype in a volume of 3 µl were added to the upper slice side. Slices, incubated with either undiluted, 1:10, or 1:100 diluted virus solutions, were cultured in the same transwell insert. After incubation for 24 h, the slices were washed three times with DPBS containing 1% P/S and transferred back to the transwell insert for continued culture. On days 1, 3, and 5 after infection, images of slices were taken as mentioned above and GFP-positive cells were counted manually to determine the number of AAV-infected cells per slice. To exclude edge effects, GFP-positive cells at the periphery of the slices were not considered. As a limitation, due to the multi-layered cell structure of the slice, the number of GFP-positive cells per slice may not be completely accurate and some extremely weak GFP-positive cells may have been overlooked during counting.

### Immunohistochemistry

Immunofluorescence analysis of AAV-infected cells was performed on day 5 after infection. Slices were fixed with 4% paraformaldehyde in DPBS for 45 min at room temperature (RT), washed three times with DPBS and incubated with 1% Triton X-100 and 1% bovine serum albumin (Roth, catalog no. T844.4) in DPBS for 2 h at RT for permeabilization and blocking of non-specific binding sites. Slices were then stained either with rabbit anti-vimentin antibody (Cell Signaling Technology, catalog no. 5741, 1:100 dilution) or mouse anti-cardiac Troponin T (cTNT) antibody (Thermo Scientific, catalog no. MS-295, 1:100 dilution) and incubated overnight at 4 °C on a plate shaker at medium speed. After washing three times with DPBS for 10 min each, the slices were incubated with the secondary antibodies Alexa Fluor 647-labeled donkey anti-rabbit IgG (Invitrogen, catalog no. A-31573, 1:200 dilution) or Alexa Fluor 647-labeled donkey anti-mouse IgG (Invitrogen, catalog no. A-31571, 1:200 dilution) for 2 h at RT in the dark on a plate shaker. The slices were washed three times in DPBS for 10 minutes each and incubated with 300 nM DAPI (Invitrogen, catalog no. D1306) in DPBS for 30 min at RT on a rocker to stain cell nuclei. Confocal images were taken using an Opera Phenix High-Content Screening system (PerkinElmer) equipped with Harmony software (version 4.9) and analyzed by Columbus software (version 2.9.1). Brightness and contrast of images were adjusted using Adobe Photoshop CC 2018.

### Statistical analysis

GraphPad Prism (version 8) was used for performing data analysis and generating graphs. Statistical significance was determined by one-way analysis of variance followed by Tukey´s post-hoc test. A p-value of less than 0.05 was considered significant.

## Results

### Characteristics and viability of organotypic heart slices

**Fig. 1:**
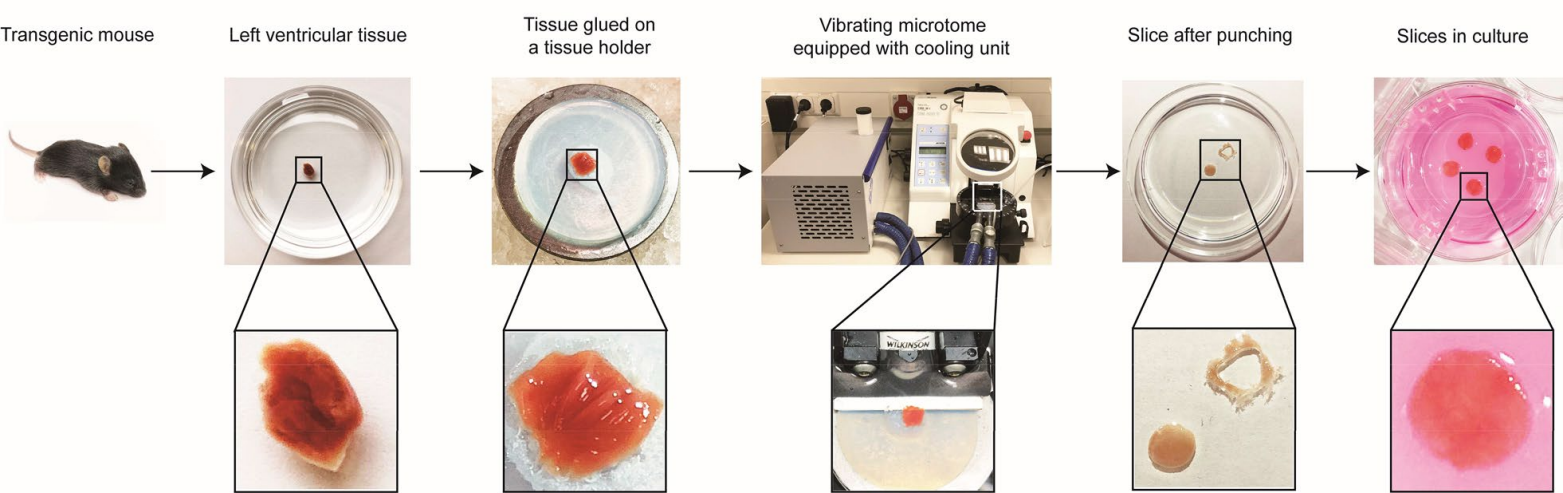
Preparation of organotypic heart slices of mouse LV myocardium. LV tissue was isolated from a transgenic homozygous αMHC-mCherry mouse line and a vibrating microtome was used to obtain sections of 300 µm thickness. Slices cut in parallel to the epicardial surface were punched using a 4 mm-diameter biopsy punch and cultured at the air-liquid interface on cell culture inserts

To establish organotypic heart slices as model system for studying AAV-mediated gene transfer into cardiac cells, we prepared myocardial slices of LV tissue from hearts of transgenic homozygous αMHC-mCherry mice using a vibrating microtome and cultured them in vitro under air-liquid interface culture conditions; a flow chart of the process is outlined in Fig. 1. Since it was anticipated from the literature that the αMHC promoter-enhancer drives cardiac-specific gene expression [34], we first tested whether mCherry expression is indeed indicative of CMs in our model. For that purpose, we stained heart slices for cTNT, a specific CM marker, and confirmed co-expression with mCherry (Additional file 1: Figure S1). To determine an optimal slice thickness for subsequent experiments, we tested several values ranging from 100–300 µm. Additional file 2: Figures S2a, b illustrate that slices with a thickness of 300 µm had a stronger mCherry signal compared to slices with 100 and 200 µm, which was attributed to the thicker slice consisting of more CMs. By performing the MTS assay, we also found that slice thickness correlated with formazan signals representing the metabolic activity of live cells and 300-µm-thick slices had the highest values (Additional file 2: Figure S2c). For these reasons, and in addition to the fact that thicker slices are easier to handle, we decided to use 300-µm-thick slices in this study. Slices thicker than 300 µm were not tested because they have been reported to suffer from poor oxygenation [35].

**Fig. 2:**
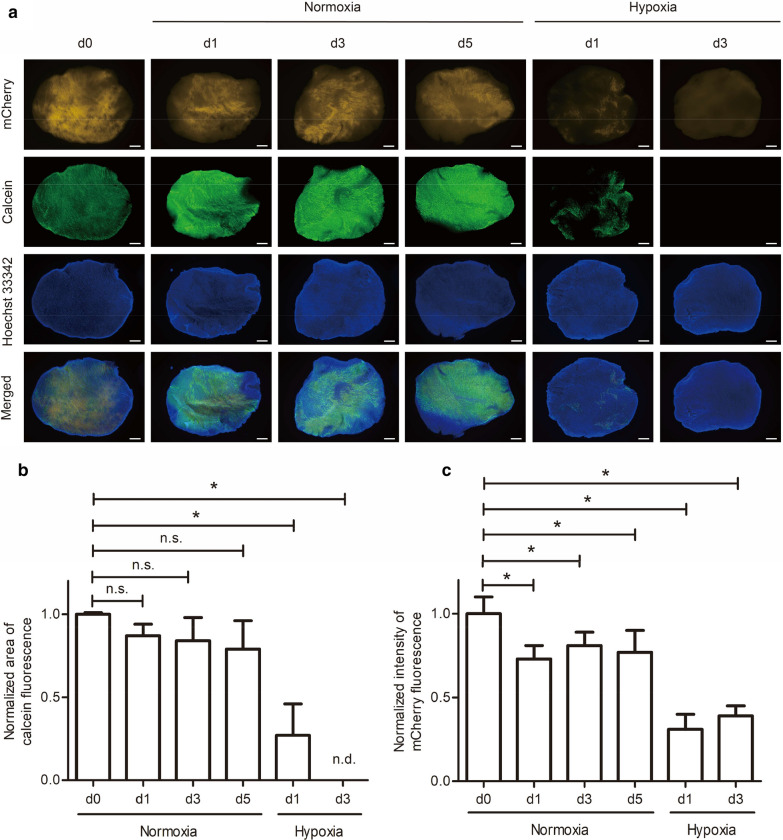
Viability of myocardial slices during culture at the air-liquid interface. **a** Heart slice viability was assessed based on mCherry fluorescence intensity and the area of calcein-stained cells for up to 5 days (d0–d5) under normoxic (20% oxygen) and hypoxic (1% oxygen) conditions. Scale bars, 500 µm. **b** Slice viability was quantified by measuring the area of calcein-stained cells on both sides of the slices on days 1–5 of culture and by normalizing to the area of day 0. **c** Survival of CMs under normoxic and hypoxic conditions was assessed by mCherry signal intensity on both sides of the slices on days 1–5 of culture normalized to day 0. In **b **and **c**, signals of three independent experiments with two to three slices each (in total, n = 6 for normoxia, n = 9 for hypoxia) were quantified, and means and SDs (standard deviations; error bars) are given. Stars indicate significant changes with p less than 0.05. n.s. indicates not significant and n.d. indicates not detected

In the following experiments, we aimed to assess the viability of 300-µm-thick slices in culture using the calcein-AM assay and found that resident cells maintained their viability in culture for at least 5 days under normoxic conditions (Fig. 2a, b). Notably, CM survival was reduced by approximately 25% after 1 day of culture, but remained unchanged for the next 4 days (Fig. 2a, c), indicating that CMs were initially sensitive to the transition from in vivo to in vitro conditions, but thereafter adjusted to the new environment. Overall, our data indicate that myocardial slices can be kept in culture for at least 5 days without apparent loss in viability and changes in tissue morphology. To mimic acute myocardial infarction, heart slices were cultured under hypoxia. We found that slice viability was significantly reduced and most cells died after 3 days in hypoxic conditions (Fig. 2a–c), indicating that in vitro a high oxygen concentration of 20% is required to maintain heart slice viability.

### Gene transfer efficiency, penetration depth, and cytotoxicity of AAV1, 2, 6, and 8

Having demonstrated that myocardial slices can be kept in culture for at least 5 days, we aimed to identify an ideal AAV serotype for efficient gene transfer into cardiac cells. For this purpose, organotypic heart slices were incubated with recombinant AAV1, 2, 6, and 8 driving GFP expression. All AAV serotypes were added directly to the upper surface of the slice to study their gene transfer efficiency. This procedure has been proposed by Griffin et al. [36] because it is a more direct approach and requires fewer virus particles than adding the virus to the culture medium.

**Fig. 3:**
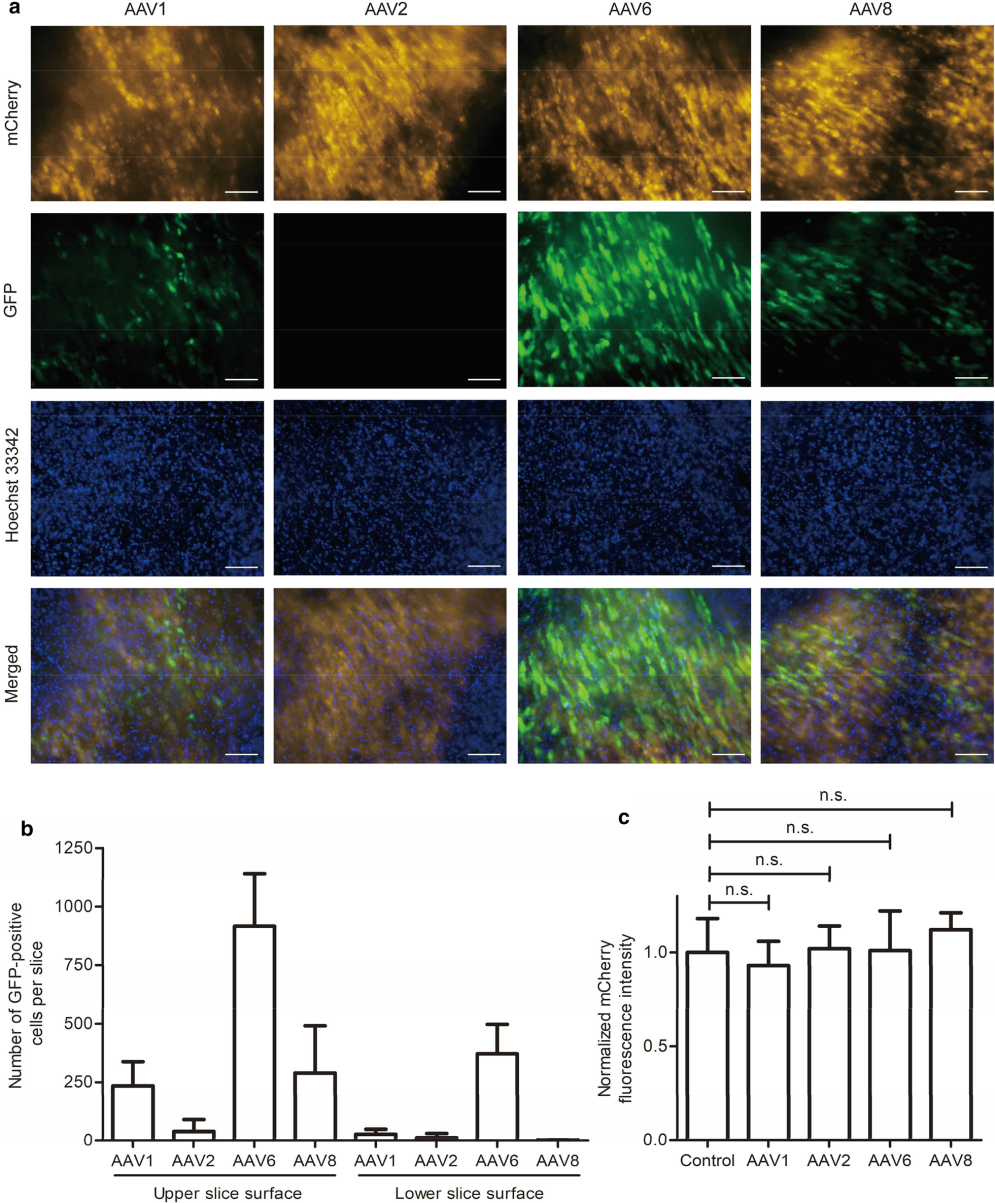
Gene transfer efficiency, penetration depth, and cytotoxicity of AAV1, 2, 6, and 8. **a** Myocardial slices were infected with four different AAV serotypes. After 5 days, slices were stained with Hoechst 33342 to label nuclei. AAV-mediated GFP expression was analyzed by fluorescence microscopy; mCherry-positive cells indicate CMs. The images shown represent the upper slice side. Scale bars, 100 µm. **b** Enumeration of GFP-positive cells per slice. Images were taken from both the upper and the lower slice side on day 5 after infection and GFP-positive cells were counted. **c** AAV cytotoxicity. mCherry signal was measured on day 5 after AAV infection on both slice surfaces and normalized to the mCherry fluorescence intensity of slices without virus (control). In **b** and **c**, signals of three independent experiments with nine slices in total were quantified, and means and SDs (error bars) are given. n.s. indicates not significant

Gene transfer efficiency was assessed by counting the number of GFP-positive cells on day 5 after infection. This time point was chosen to account for the possibility of varying gene expression onset among the different AAV serotypes tested. We found that AAV6 had the highest gene transfer efficiency in heart slices (Fig. 3a). Heart slices incubated with AAV1 and AAV8 showed fewer GFP-positive cells compared to AAV6, whereas almost no GFP-expressing cells were detected after incubation with AAV2. Furthermore, we studied virus penetration through heart slices by enumerating GFP-positive cells on the upper and the lower surface of the slice. The addition of AAV6 to the top of the slice led to a high number of GFP-positive cells on the bottom of the slice, whereas the addition of the other three AAV serotypes resulted in very few GFP-positive cells on the bottom side (Fig. 3b). To investigate whether the viruses were cytotoxic to CMs at the concentration used, we compared the mCherry fluorescence intensity of virus-infected slices to non-infected control slices on day 5. For all four AAV serotypes tested, no significant difference was found, indicating that, at least for CMs, the doses were non-toxic (Fig. 3c). In sum, these data demonstrate that AAV6 has the highest gene transfer efficiency and penetration rate into cardiac cells without exerting cytotoxic effects.

### Infiltration of AAV6 into heart slices

**Fig. 4:**
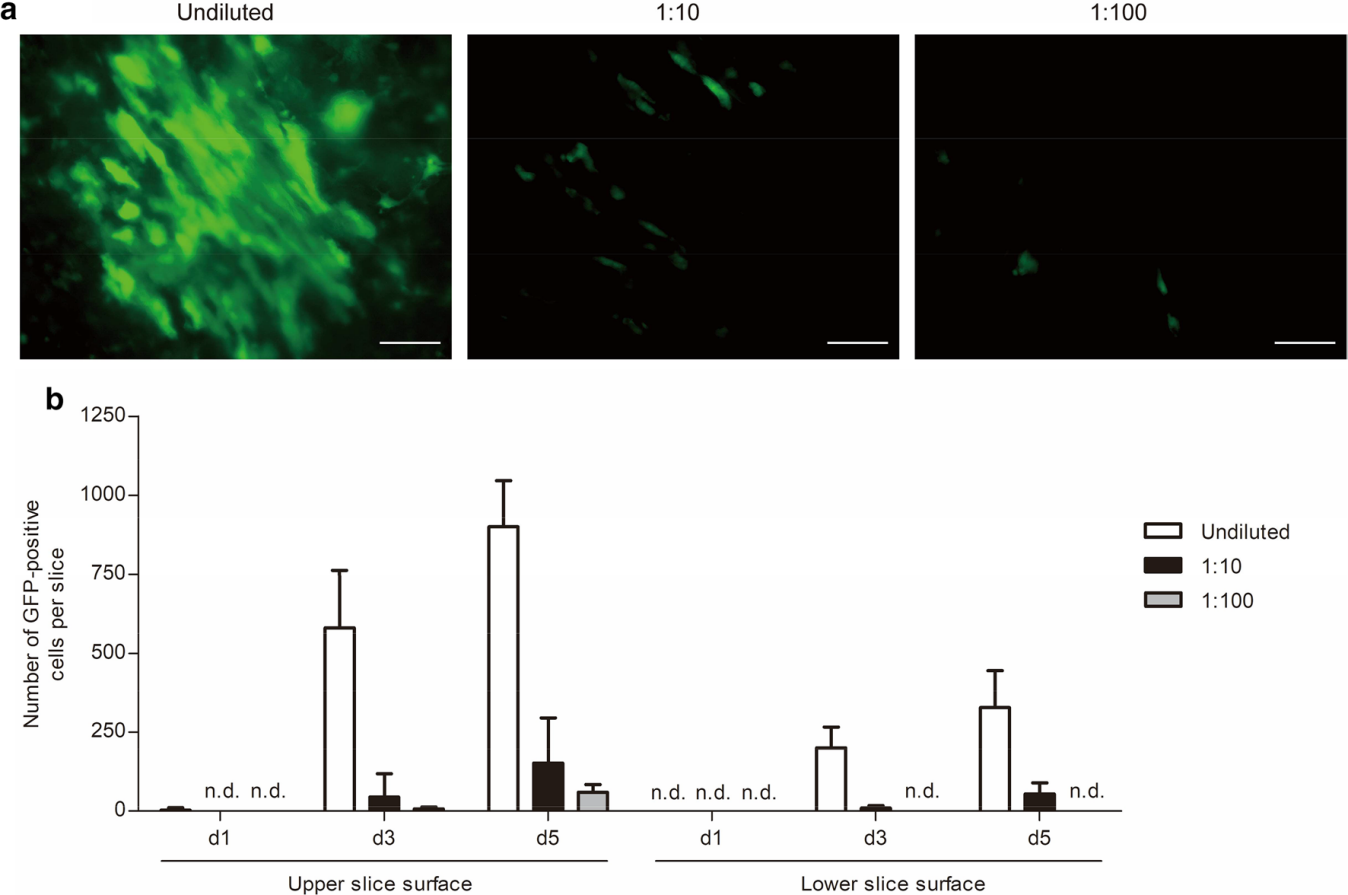
AAV6 dose-dependent GFP expression kinetics in organotypic heart slices. **a** Myocardial slices were incubated with either undiluted (4 × 10^8^ vector genomes), 1:10, or 1:100 diluted virus solutions. AAV6-mediated GFP expression was analyzed by fluorescence microscopy on day 5 after infection. Images were taken from the upper slice side. Scale bars, 100 µm. **b** Quantification of time-course and concentration-dependent gene transfer efficiency of AAV6. GFP-positive cells were counted on both the upper and lower slice surface on days 1, 3, and 5 after AAV infection. Three independent experiments with six slices in total were quantified, and means and SDs (error bars) are given. n.d. indicates not detected

Having determined AAV6 as an efficient gene transfer vector for cardiac cells, we decided to investigate its infiltration into heart slices in more detail by analyzing gene expression kinetics and dose-response relationships. For this purpose, heart slices were incubated with either undiluted, 1:10, or 1:100 diluted AAV6 solutions. GFP expression was analyzed on days 1, 3, and 5 after infection by fluorescence microscopy and gene transfer was quantified by subsequent image analysis. GFP expression was concentration-dependent with a notable dose-response relationship (Fig. 4a). When comparing the upper and the lower surface of slices over time, we found an increase of GFP-positive cells per slice on both sides, starting with almost no GFP-positive cells on day 1 and resulting in approximately 900 GFP-positive cells on the upper slice side on day 5 after incubation with undiluted AAV6 solution (Fig. 4b). Similarly, more GFP-positive cells were found over time for the 1:10 and 1:100 AAV6 dilutions on the upper slice side. However, after incubation with 1:10 and 1:100 diluted virus solutions, we found almost no GFP-positive cells on the lower slice surface, indicating that a certain virus load is required for effective AAV6 infiltration of heart slices within 5 days (Fig. 4b). In sum, a minimum concentration of 4 × 10^8^ AAV6 vector genomes per slice (approximately 1 × 10^8^ AAV6 vector genomes per 1 mm^3^ of heart tissue) was needed to ensure a strong virus infiltration through 300-µm-thick organotypic heart slices.

### Cell tropism of AAV6

**Fig. 5:**
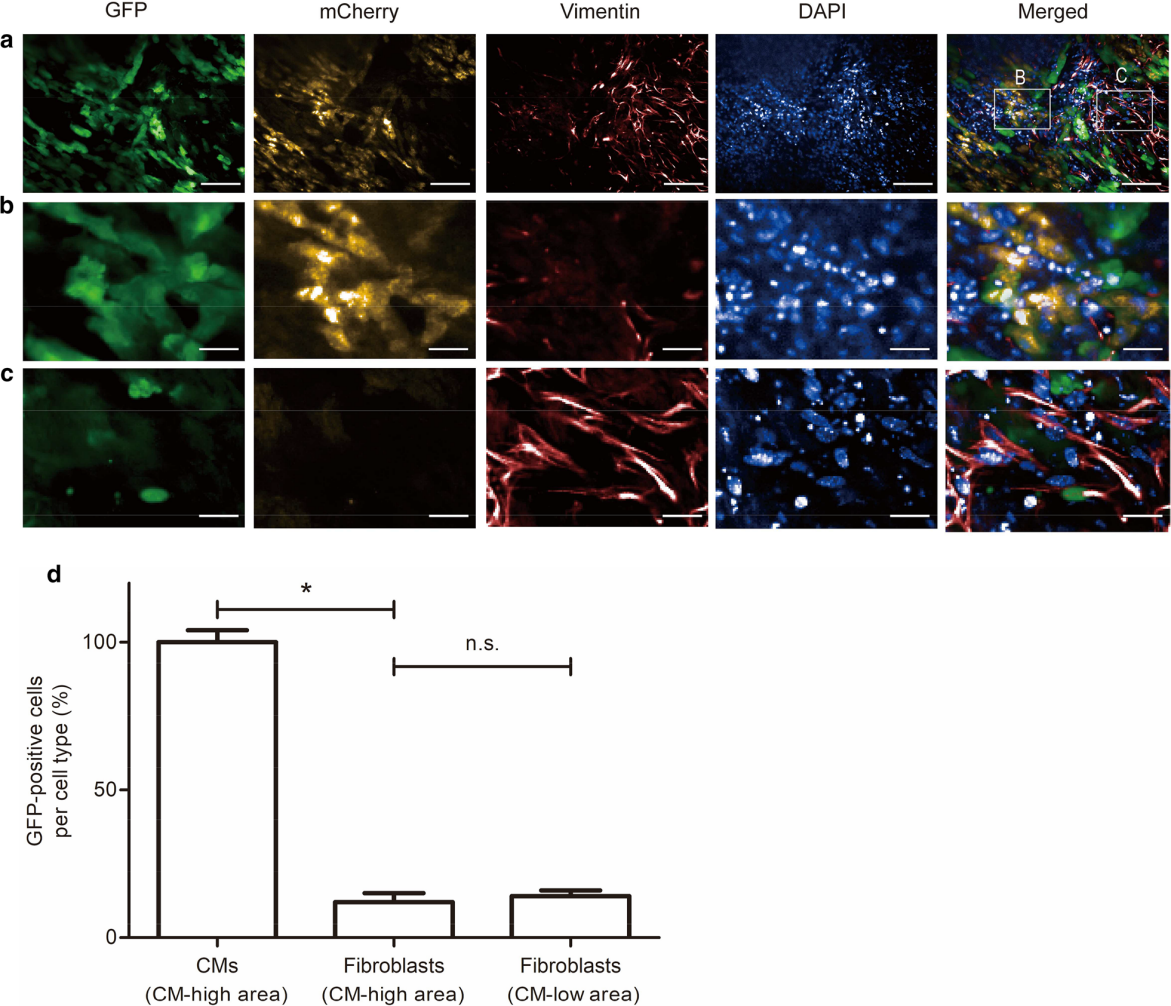
AAV6 cell tropism in organotypic heart slices. **a** To investigate AAV6-mediated GFP expression in CMs and fibroblasts, heart slices were infected with AAV6 and, on day 5, were probed with an antibody directed against vimentin and stained with DAPI, and were analyzed by confocal fluorescence microscopy. GFP-positive cells represent virus-infected cells, mCherry-positive cells indicate CMs and vimentin-positive cells indicate fibroblasts. **b** Enlarged CM-high area with tightly packed CMs and no apparent gaps between single cells. **c** Enlarged CM-low area with fewer CMs but a higher density of fibroblasts. **d** Percentage of GFP-positive cells per cell type. Twenty-five CMs and 25 fibroblasts with high mCherry and vimentin signals, respectively, were counted and analyzed for GFP expression. Four slices distributed over three independent experiments were quantified, and means and SDs (error bars) are given. A star indicates a significant change with p less than 0.05. n.s. indicates not significant. Scale bars, 100 µm (**a**) and 25 µm (**b**, **c**)

To analyze AAV6 cell tropism for specific heart cells, we added the virus to myocardial slices and analyzed GFP expression in CMs and cardiac fibroblasts. Both cell types were readily distinguishable based on cell size and morphology. CMs exhibit a characteristic rod-shaped morphology, whereas fibroblasts have a spindle-shaped morphology and are smaller in size (Fig. 5a). Intriguingly, we found regions where CMs showed bright mCherry fluorescence and were close to each other with no apparent gaps (defined as CM-high area, Fig. 5b), and other regions with fewer CMs and more fibroblasts (defined as CM-low area, Fig. 5c). CMs and fibroblasts were analyzed for GFP expression in the CM-high area 5 days after infection and we found that the proportion of GFP-positive CMs was approximately eightfold higher than that of GFP-positive fibroblasts (Fig. 5d). Interestingly, we saw no significant difference between the fractions of GFP-positive fibroblasts in CM-high or CM-low regions. The efficiency of AAV6-mediated gene transfer into CMs in the CM-low area could not be assessed due to an insufficient number of CMs. In sum, the data indicate that AAV6 has a strong preference for infecting CMs and that its low gene transfer efficiency into fibroblasts is independent of the local tissue structure and/or microenvironment.

## Discussion

Gene therapy represents a promising avenue for the treatment of HF, but its clinical application still requires further investigation. In this study, we have established an organotypic heart slice culture model using LV tissue from approximately 2-week-old transgenic mice and used this platform to analyze AAV serotype-dependent gene transfer into cardiac tissue. To the best of our knowledge, this is the first report of AAV-mediated gene delivery in living myocardial slices.

Our organotypic heart slice model enables a more meaningful examination of potential gene therapy vectors due to the combination of the simplicity and accessibility of two-dimensional in vitro culture with the advantages of three-dimensional tissue complexity, which includes multiple cardiac cell types embedded in a three-dimensional extracellular matrix network mimicking in vivo heart physiology. The observed overall high viability of mouse myocardial slices during 5-day culture is consistent with other studies in small and large mammals, such as rodents, dogs, and humans [29, 31]. In contrast, Ou et al. reported that the viability of slices from pig hearts decreased significantly after 2 days in culture [32]; however, this could be explained by the permanent presence of 2,3-butanedione monoxime in the culture medium, which is known to cause cell damage over time [37]. It has recently been demonstrated that mechanical load and electrical stimulation are important for maintaining structural and functional properties during long-term culture of organotypic heart slices [38–40]. Our model does not include mechanical or electrical stimulation, and we acknowledge this limitation. Our future research efforts will focus on model development to address these issues accordingly.

Notably, in our heart slices we found regions with tightly packed CMs and regions with fewer CMs and a high density of fibroblasts, a characteristic of ischemically injured heart tissue [41], which likely resulted from damage during slice preparation or culture. These slices potentially provide an insight into the situation in infarcted and fibrotic tissues, where the CM number is reduced, and conceptually allow to test in situ gene transfer approaches for the treatment of ischemic heart disease. These CM-low regions might also be useful for studying other repair approaches using cell replacement, protein, or pharmacological therapy. Additionally, the reduction in viability of heart slices cultured under 1% oxygen makes them a potentially suitable model for examining the acute phase of ischemic heart damage when combined with glucose deprivation. In future studies, heart slices could be cultured under hypoxia for shorter time periods or under higher oxygen concentrations (> 1%) to provide an appropriate testing environment for myocardial regeneration approaches.

After establishing a protocol for the culture of myocardial slices, gene transfer efficiency, expression kinetics, penetration depth, and cell-type tropism were evaluated for AAV1, 2, 6, and 8 based on AAV-mediated GFP expression. The addition of AAV6 to heart slices resulted in the highest number of GFP-positive cells, almost exclusively CMs. In contrast, slice incubation with AAV1, 2, and 8 led to fewer GFP-expressing cells, with AAV2 having the lowest gene transfer efficiency, agreeing with previous results showing that AAV2 induced gene expression relatively slowly in the heart [22]. Thus, our results indicate that AAV6 is a superior candidate for gene delivery into CMs. Moreover, AAV6 showed the deepest tissue penetration of the four serotypes tested, however a 300-µm-thick heart slice does not reflect the clinical setting, because in the human heart the LV wall alone is several centimeters thick [31]. Therefore, methods for increasing the penetration depth are an important issue that must be considered in the future.

Although AVV tissue and cell tropism have been previously examined both in in vivo models and in monocellular in vitro cultures [5, 25, 42, 43], cell type-specific gene transfer by AAV in heart tissue has not been investigated. In this study, we show for the first time that AAV6 predominantly infects CMs rather than cardiac fibroblasts. It is not yet known whether and to what extent resident endothelial and smooth muscle cells are also targeted by AAV6, but these aspects should be the subject of future investigation. Modification of AAV6 cell tropism by transcriptional or transductional targeting using cell-specific promoters or capsid modifications, respectively, could be applied to restrict infection to CMs and avoid potential negative off-target effects [6, 19, 44].

Finally, we tested the cytotoxicity of all four AAV serotypes and found no signs of toxicity at the cellular level in CMs 5 days after AAV infection, which is consistent with the results in cell monolayer studies [43]. Of note, the AAV concentration used in our study is comparable to the doses used for systemic AAV injection in mice [5]. Thus, in conjunction with studies reporting a low genotoxicity profile [45], weak cytotoxic T-lymphocyte-mediated toxicity [46], and no impairment of heart function after AAV injection [5, 42], our data suggest that an AAV dose of approximately 10^8^ vector genomes per mm^3^ tissue could be considered safe.

## Conclusions

In conclusion, we have established a myocardial organotypic culture model and found that AAV6 has the highest gene transfer efficiency into CMs. This knowledge will help to develop more effective gene delivery strategies using AAV vectors specifically targeting CMs. Future research should focus on determining and minimizing off-target effects in non-myocytes, as well as examining the transfer of therapeutic genes to diseased heart slices.

## Supplementary information

**Additional file 1. **Verification of co-expression of mCherry and cTNT in transgenic heart slices. Heart slices were probed on day 5 with an antibody directed against cTNT, stained with DAPI, and were analyzed by fluorescence microscopy. Three images representing a 10-µm-thick Z-stack were merged to a single image. Scale bars, 20 µm.

**Additional file 2.** Validation of mCherry fluorescence intensity as indicator for the number of live CMs present in heart slices of varying thickness. **a** Slices with a thickness of 100–300 µm were analyzed on day 0 for CM-specific mCherry expression by fluorescence microscopy. Notably, mCherry fluorescence intensity was not evenly distributed across the slice, which could be explained by mechanical damage occurring during the flattening of the originally curved LV cavity. Scale bars, 500 µm. **b** Quantification of mCherry fluorescence intensity normalized to that of 300-µm-thick slices. **c** Absorbance of formazan normalized to that of 300-µm-thick slices. In **b** and **c**, signals of three independent experiments with ten slices in total were quantified, and means and SDs (error bars) are given. Stars indicate significant changes with p less than 0.05.

## Data Availability

The datasets used and/or analyzed during the current study are available from the corresponding author on reasonable request.

## Abbreviations

AAV: Adeno-associated virus
calcein-AM: Calcein-acetoxymethyl ester
CM: Cardiomyocyte
cTNT: Cardiac Troponin T
DPBS: Dulbecco's phosphate-buffered saline with magnesium and calcium
GFP: Green fluorescent protein
HF: Heart failure
LV: Left ventricular
MTS: 3-(4,5-Dimethylthiazol-2-yl)-5-(3-carboxymethoxyphenyl)-2-(4-sulfophenyl)-2H-tetrazolium
P/S: Penicillin/streptomycin
RT: Room temperature
SD: Standard deviation
αMHC: Alpha-myosin heavy-chain
