# Supplementary figures and images for: Comparative analysis of adeno-associated virus serotypes for gene transfer in organotypic heart slices
Source: J Transl Med. 2020 Nov 18;18:437. doi: 10.1186/s12967-020-02605-4 (PMC7673099; doi:10.1186/s12967-020-02605-4)

mCherry

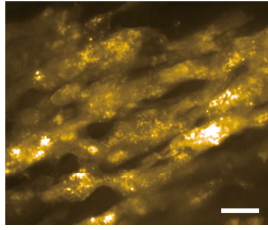

cTNT

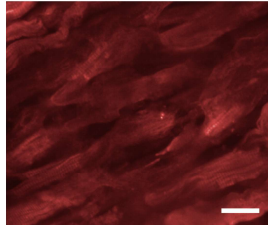

DAPI

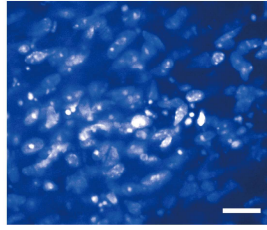

Merged

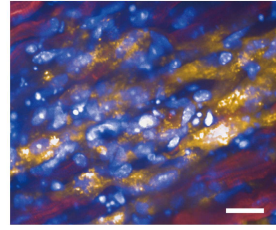

Supplement: Supplementary file 1 — Additional file 1. Verification of co-expression of mCherry and cTNT in transgenic heart slices. Heart slices were probed on day 5 with an antibody directed against cTNT, stained with DAPI, and were analyzed by fluorescence microscopy. Three images representing a 10-µm-thick Z-stack were merged to a single image. Scale bars, 20 µm. [file 12967_2020_2605_MOESM1_ESM.pdf]

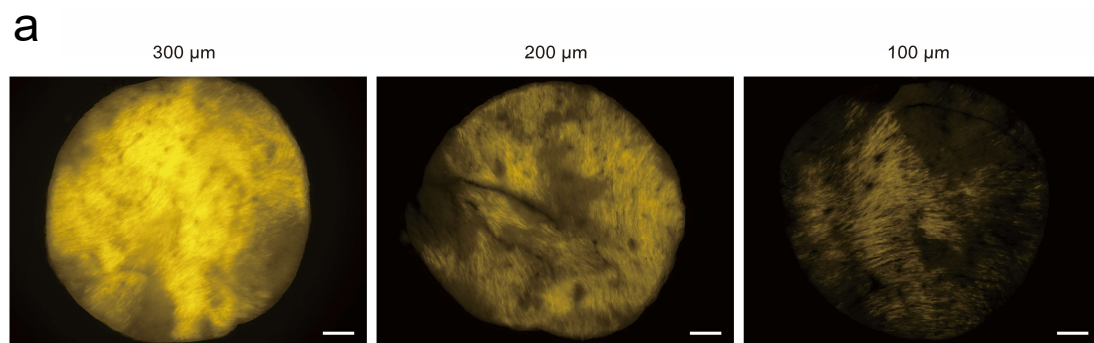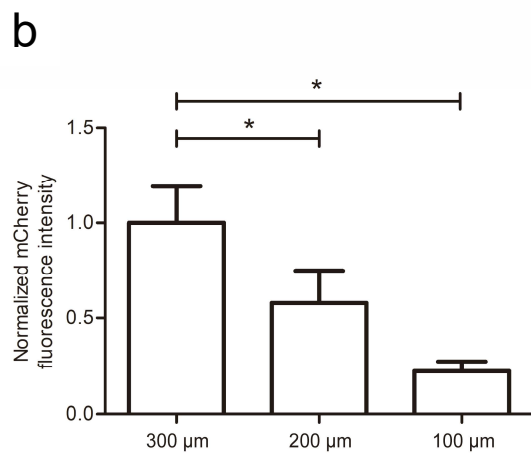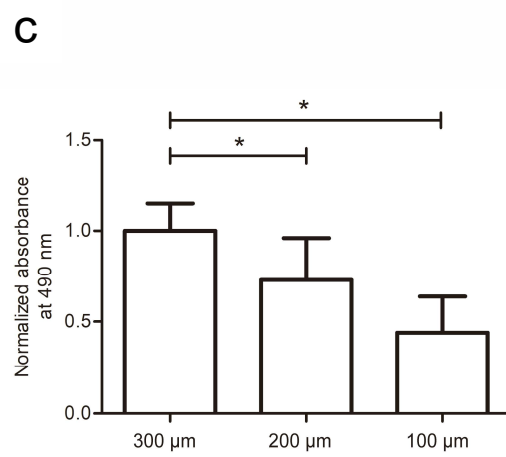

Supplement: Supplementary file 2 — Additional file 2. Validation of mCherry fluorescence intensity as indicator for the number of live CMs present in heart slices of varying thickness. a Slices with a thickness of 100–300 µm were analyzed on day 0 for CM-specific mCherry expression by fluorescence microscopy. Notably, mCherry fluorescence intensity was not evenly distributed across the slice, which could be explained by mechanical damage occurring during the flattening of the originally curved LV cavity. Scale bars, 500 µm. b Quantification of mCherry fluorescence intensity normalized to that of 300-µm-thick slices. c Absorbance of formazan normalized to that of 300-µm-thick slices. In b and c, signals of three independent experiments with ten slices in total were quantified, and means and SDs (error bars) are given. Stars indicate significant changes with p less than 0.05. [file 12967_2020_2605_MOESM2_ESM.pdf]
